# Comparison of Survival and Patterns of Recurrence in Gastric Neuroendocrine Carcinoma, Mixed Adenoneuroendocrine Carcinoma, and Adenocarcinoma

**DOI:** 10.1001/jamanetworkopen.2021.14180

**Published:** 2021-07-27

**Authors:** Jianxian Lin, Yajun Zhao, Yanbing Zhou, Yantao Tian, Qingliang He, Junpeng Lin, Hankun Hao, Bingbing Zou, Lixin Jiang, Gang Zhao, Wei Lin, Yanchang Xu, Zhi Li, Fangqin Xue, Shuliang Li, Weihua Fu, Yongxiang Li, Zekuan Xu, Yong Li, Jinping Chen, Xiaojun Zhou, Zhenggang Zhu, Lisheng Cai, En Li, Honglang Li, Chaohui Zheng, Ping Li, Changming Huang, Jianwei Xie

**Affiliations:** 1 Department of Gastric Surgery, Fujian Medical University Union Hospital, Fuzhou, China; 2 Key Laboratory of Ministry of Education of Gastrointestinal Cancer, Fujian Medical University, Fuzhou, China; 3 Department of Gastrointestinal Surgery, West District, First Affiliated Hospital of the University of Science and Technology of China, Division of Life Sciences and Medicine, University of Science and Technology of China, Hefei, China; 4 Department of Gastrointestinal surgery, Affiliated Hospital of Qingdao University, Qingdao, China; 5 Department of Pancreatic and Gastric Surgery, National Cancer Center, National Clinical Research Center for Cancer, Cancer Hospital, Chinese Academy of Medical Sciences and Peking Union Medical College, Beijing, China; 6 Department of gastrointestinal surgery, the First Affiliated Hospital of Fujian Medical University, Fuzhou, Fujian Province, China; 7 Department of General Surgery, Huashan Hospital, Fudan University, Shanghai, China; 8 Department of Gastrointestinal Surgery, First Affiliated Hospital of Anhui Medical University, Hefei, China; 9 Department of Gastrointestinal Surgery, Yantai Yuhuangding Hospital, Yantai, China; 10 Department of Gastrointestinal Surgery, Renji Hospital, Shanghai Jiaotong University, Shanghai, China; 11 Department of Gastrointestinal Surgery, Gastrointestinal Surgery Research Institute, Affiliated Hospital of Putian University, Putian, China; 12 Department of Gastrointestinal Surgery, Fujian Medicine University Teaching Hospital, First Hospital of Putian, Putian, China; 13 Department of General Surgery, Henan Cancer Hospital, Zhengzhou, China; 14 Department of Gastrointestinal Surgery, Provincial Clinical Medical College of Fujian Medical University, Fujian Provincial Hospital, Fuzhou, China; 15 Department of Gastrointestinal Surgery, Second People’s Hospital of Liaocheng, Liaocheng, China; 16 Department of General Surgery, Tianjin Medical University General Hospital, Tianjin, China; 17 Department of General Surgery, First Affiliated Hospital of Nanjing Medical University, Nanjing, China; 18 Department of General Surgery, Guangdong Provincial People’s Hospital, Guangdong Academy of Medical Sciences, Guangzhou, China; 19 Department of Gastrointestinal Surgery, Quanzhou First Hospital Affiliated to Fujian Medical University, Quanzhou, China; 20 Department of Gastroenterology Surgery, First Affiliated Hospital of Soochow University, Suzhou, China; 21 Department of Gastrointestinal Surgery, Ruijin Hospital Affiliated to Shanghai Jiao Tong University School of Medicine, Shanghai, China; 22 Department of General Surgery, Zhangzhou Affiliated Hospital of Fujian Medical University, Zhangzhou, China; 23 Department of Gastrointestinal Surgery, Meizhou People’s Hospital, Meizhou, China; 24 Department of Gastrointestinal Surgery, Second Affiliated Hospital, Nanchang University, Nanchang, China

## Abstract

**Importance:**

Gastric neuroendocrine carcinoma and mixed adenoneuroendocrine carcinoma are rare pathological types of gastric cancer, and there is a lack of multicenter studies comparing the prognosis and recurrence patterns of gastric neuroendocrine carcinoma, gastric mixed adenoneuroendocrine carcinoma, and gastric adenocarcinoma.

**Objective:**

To compare the differences in long-term survival and patterns of recurrence among gastric neuroendocrine carcinoma, gastric mixed adenoneuroendocrine carcinoma, and gastric adenocarcinoma.

**Design, Setting, and Participants:**

This cohort study included patients with resectable gastric neuroendocrine carcinoma and gastric mixed adenoneuroendocrine carcinoma at 23 hospitals in China from January 2006 to December 2016. In addition, patients with gastric adenocarcinoma were selected as controls. Propensity score–matched analysis was used to match pathological stage among the different pathological types, and disease-free survival (DFS), postrecurrence survival (PRS), and patterns of recurrence were examined. Data analysis was conducted from July 15, 2020, to October 21, 2020.

**Exposures:**

Curative resection for gastric neuroendocrine carcinoma, gastric mixed adenoneuroendocrine carcinoma, and gastric adenocarcinoma.

**Main Outcomes and Measures:**

The main outcomes were DFS and patterns of recurrence.

**Results:**

A total of 3689 patients were analyzed (median [interquartile range] age, 62 [55-69] years; 2748 [74.5%] men), including 503 patients (13.6%) with gastric neuroendocrine carcinoma, 401 patients (10.9%) with gastric mixed adenoneuroendocrine carcinoma, and 2785 patients (75.5%) with gastric adenocarcinoma. After propensity score matching, 5-year DFS was 47.6% (95% CI, 42.7%-52.5%) for patients with gastric neuroendocrine carcinoma, compared with 57.6% (95% CI, 55.1%-60.1%) with gastric adenocarcinoma (*P* < .001) and 51.1% (95% CI, 46.0%-56.2%) for patients with gastric mixed adenoneuroendocrine carcinoma, compared with 57.8% (95% CI, 55.1%-60.5%) patients with gastric adenocarcinoma (*P* = .02). Multivariable analyses found that, compared with gastric adenocarcinoma, gastric neuroendocrine carcinoma (hazard ratio [HR], 1.64; 95% CI, 1.40-1.93) and gastric mixed adenoneuroendocrine carcinoma (HR, 1.25; 95% CI, 1.05-1.49) were independent risk factors associated with worse DFS. Compared with matched patients with gastric adenocarcinoma, patients with gastric neuroendocrine carcinoma were more likely to have distant recurrence (268 patients [17.2%] vs 101 patients [23.7%]; *P* = .002), as were patients with gastric mixed adenoneuroendocrine carcinoma (232 patients [17.3%] vs 76 patients [22.8%]; *P* = .02). In multivariate analysis, gastric neuroendocrine carcinoma (HR, 2.22; 95% CI, 1.66-2.98) and gastric mixed adenoneuroendocrine carcinoma (HR, 1.70; 95% CI, 1.24-2.34) were independent risk factors associated with distant recurrence. Additionally, T3 to T4 stage (odds ratio, 2.84; 95% CI, 1.57-5.14; *P* = .001) and lymph node metastasis (odds ratio, 2.01; 95% CI, 1.31-3.10; *P* = .002) were independent risk factors associated with distant recurrence of gastric neuroendocrine carcinoma and gastric mixed adenoneuroendocrine carcinoma.

**Conclusions and Relevance:**

This cohort study found that patients with gastric neuroendocrine carcinoma or gastric mixed adenoneuroendocrine carcinoma had worse prognoses and were more prone to distant recurrence than those with gastric adenocarcinoma. Thus, different follow-up and treatment strategies should be developed to improve the long-term survival of patients with gastric neuroendocrine carcinoma or gastric mixed adenoneuroendocrine carcinoma, especially patients with tumors penetrating into the subserosa or deeper layers or with lymph node metastasis.

## Key Points

**Question:**

Are there any differences in prognoses or recurrence patterns associated with gastric neuroendocrine carcinoma, mixed adenoneuroendocrine carcinoma, or adenocarcinoma?

**Findings:**

This cohort study included 3689 patients with resectable gastric adenocarcinoma, gastric neuroendocrine carcinoma, or gastric mixed adenoneuroendocrine carcinoma. Propensity score matching analysis found that, compared with gastric adenocarcinoma, gastric neuroendocrine carcinoma and gastric mixed adenoneuroendocrine carcinoma were independent risk factors associated with worse disease-free survival, postrecurrence survival, and distant recurrence outcomes.

**Meaning:**

These findings suggest that different follow-up and treatment strategies should be developed to improve the long-term survival of patients with gastric neuroendocrine carcinoma or gastric mixed adenoneuroendocrine carcinoma, especially for patients with tumors penetrating into the subserosa or deeper layers and with lymph node metastasis.

## Teaser

This cohort study examines differences in long-term survival and patterns of recurrence among patients with gastric neuroendocrine carcinoma, gastric mixed adenoneuroendocrine carcinoma, and gastric adenocarcinoma.

## Introduction

Gastric cancer, as a common cancer, is one of the main causes of cancer-related deaths worldwide,^1^ and adenocarcinoma is a common pathological type. However, gastric neuroendocrine neoplasm is a rare type of gastric cancer. In 2011, the World Health Organization (WHO)^2^ classified neuroendocrine neoplasms of the stomach into 3 categories: neuroendocrine tumor, neuroendocrine carcinoma, and mixed adenoneuroendocrine carcinoma, in which gastric neuroendocrine carcinoma consists of poorly differentiated, high-grade malignant neoplasms, while gastric mixed adenoneuroendocrine carcinoma contains at least 30% each of epithelial and neuroendocrine components. As a highly invasive tumor, gastric neuroendocrine carcinoma tends to present in late stages and to be associated with lymph node metastasis.^3,4^ Previous studies have shown that the prognoses for gastric neuroendocrine carcinoma and gastric mixed adenoneuroendocrine carcinoma are worse than that of gastric adenocarcinoma, but most of those studies were single-center studies with small sample sizes.^5,6,7^

Currently, follow-up strategies for gastric neuroendocrine carcinoma and gastric mixed adenoneuroendocrine carcinoma are similar to those for gastric adenocarcinoma.^8^ Understanding the patterns of recurrence is important in designing follow-up and treatment strategies.^9,10^ However, previous studies on the recurrence pattern of gastric cancer have mainly focused on gastric adenocarcinoma.^10,11,12^ The patterns of recurrence and postrecurrence survival for patients with gastric neuroendocrine carcinoma or gastric mixed adenoneuroendocrine carcinoma are still unknown and have not been compared with those of patients with gastric adenocarcinoma, to our knowledge. The purpose of this study was to compare the survival and recurrence patterns among patients with resectable gastric neuroendocrine carcinoma, gastric mixed adenoneuroendocrine carcinoma, or gastric adenocarcinoma by using a multicenter, large sample series.

## Methods

This study was performed in accordance with the Declaration of Helsinki^13^ and the Ethical Guidelines for Clinical Studies, and was approved by the institutional review boards of all participating centers. Written informed consent or a substitute (eg, assent from a caretaker) for it was obtained from all patients for inclusion in the study. This study followed the Strengthening the Reporting of Observational Studies in Epidemiology (STROBE) reporting guideline.

This multicenter retrospective cohort study was conducted in patients with gastric neuroendocrine carcinoma or gastric mixed adenoneuroendocrine carcinoma without metastatic disease who underwent potentially curative resection in 23 centers of the China Gastric Neuroendocrine Tumor Study Group from January 2006 to December 2016. All the centers included in this study are large- and medium-sized teaching hospitals in China that have rich experience in the diagnosis and treatment of gastric cancer. The annual number of gastric cancer surgeries in each center is more than 300 surgeries per year. The main exclusion criteria were patients who underwent completion gastrectomy, patients with incomplete data (including tumor size and follow-up information), and patients who experienced postoperative death within 30 days. Patients with remnant gastric cancer, unknown tumor differentiation, or unknown follow-up data and patients who died within 30 days after surgery were also excluded. Patients who died within 1 month of surgery were excluded because death within 30 days is usually not from recurrent disease but related to complications.^14^

The diagnostic criteria for gastric neuroendocrine carcinoma and gastric mixed adenoneuroendocrine carcinoma were based on the WHO 2011 classification.^2^ In each center, the postoperative pathological findings were confirmed by 2 experienced upper gastrointestinal pathologists. All surgical procedures, including D2 lymphadenectomy, were performed according to the Japanese gastric cancer treatment guidelines.^15^ Adjuvant chemotherapy was recommended for patients with advanced gastric neuroendocrine carcinoma, gastric mixed adenoneuroendocrine carcinoma, or gastric adenocarcinoma.^8,16^

### Patient Characteristics and Follow-up

Demographic and clinicopathological characteristics and treatment of the study population were determined by review of the database and of medical records. The pathological stages were reevaluated by pathologists according to the American Joint Committee on Cancer (AJCC) guidelines.^17^ The median number of patients with gastric neuroendocrine carcinoma or gastric mixed adenoneuroendocrine carcinoma in each center was used as the cutoff value to define high-volume (≥30 patients) or low-volume (<30 patients) centers.

The last follow-up time was December 2019. Postoperative follow-up was performed every 3 months for 2 years and then every 6 months in years 3 to 5, and every year for after 5 years. Most patients routinely received physical examinations, laboratory tests, chest radiography, abdominal ultrasonography or computed tomography, and an annual endoscopic examination.

### Outcomes and Pattern of Recurrence

The primary outcomes of the study were disease-free survival (DFS) and patterns of recurrence. DFS was defined as the time interval from the date of the operation to the date of recurrence or death with evidence of recurrence. Overall survival (OS) was calculated from the date of surgery to the date of last contact or death. Postrecurrence survival (PRS) was defined as the time from first recurrence to either death or the last follow-up,^12,18^ and patients who experienced recurrence and died in the same month were censored at the evaluation of PRS.

The recurrence was diagnosed with radiologic findings or biopsies of suspicious lesions during surveillance after surgery,^19^ which were recorded from the date of initial surgery to the date of the first recurrence, death of any cause, or last follow-up, whichever occurred first.^20^ As previously described,^10,21^ recurrences were categorized according to the site involved as locoregional, peritoneal, distant, or multiple. The presence of recurrent disease in 2 or more sites was categorized as multiple recurrence. Multiple recurrence in the same area (eg, anastomotic and gastric bed) was coded in a single category.

### Statistical Analysis

Propensity score matching was used to adjust the imbalance of potential confounding factors among different pathological types. Two propensity score models were generated for pairwise comparisons (including gastric neuroendocrine carcinoma vs gastric adenocarcinoma and gastric mixed adenoneuroendocrine carcinoma vs gastric adenocarcinoma). Tumor staging is an important pathological factor associated with the prognosis of gastric neuroendocrine carcinoma, gastric mixed adenoneuroendocrine carcinoma, and gastric adenocarcinoma,^22,23^ while age and sex are common clinical factors associated with the prognosis of a tumor.^24,25^ Thus, propensity scores were calculated using a logistic regression model, including pathological TNM stage, age, and sex, for all patients. Then, a 1 to 4 matching procedure according to the propensity score, without replacement, was undertaken using the nearest-neighbor method within a caliper of 0.01. Propensity score matching was performed using the matching package in R version 3.5.2 (R Project for Statistical Computing).^26^

Continuous variables are expressed as the medians and interquartile ranges (IQRs), and categorical variables are expressed as numbers and percentages. Differences among groups in categorical variables were analyzed using the χ^2^ test or Fisher exact test, whereas differences in continuous variables were evaluated using *t* tests or Mann-Whitney *U* tests. Survival rates and median survival outcomes were estimated with Kaplan-Meier curves. The log-rank test was performed for comparisons between groups. The incidence of recurrence and the different proportions of recurrent sites between 2 groups were evaluated by the χ^2^ test or Fisher exact test.^19,27^ A Cox proportional hazards regression model was used to identify the independent prognostic factors associated with DFS. The proportional hazard was evaluated by proportional hazards assumption test and Schoenfeld residual test.^28^ A logistic regression model was used to identify the risk factors associated with distant recurrence. Variables with *P* < .05 in the univariable analysis were included in the multivariable model, and forward likelihood ratio method is used for analysis.^29^ All tests were 2-sided, and statistical significance was inferred at *P* < .05. Statistical analyses were performed using SPSS statistical software version 22.0 (IBM). Data were analyzed from July 15, 2020, to October 21, 2020.

## Results

### Baseline Characteristics and Survival Outcomes From the Unmatched Data

A total of 3689 patients (median [IQR] age, 62 [55-69] years; 2748 [74.5%] men) were included in the analysis, including 503 patients with gastric neuroendocrine carcinoma (13.6%), 401 patients with gastric mixed adenoneuroendocrine carcinoma (10.9%), and 2785 patients with gastric adenocarcinoma (75.5%) (eFigure 1 in the Supplement). There were 12 high-volume centers, which treated a total of 846 patients (22.9%), and 11 low-volume centers, which treated a total of 58 patients (0.2%) (eTable 1 in the Supplement). The remaining 2785 patients (75.5%) were patients with gastric adenocarcinoma and all came from 1 center. Compared with patients with gastric adenocarcinoma, patients with gastric neuroendocrine carcinoma or gastric mixed adenoneuroendocrine carcinoma patients were older (median [IQR] age, 61 [54-68] years vs 64 [58-69] and 64 [58-70] years), more likely to be men (2035 [73.1%] men vs 400 men [79.5%] and 313 [78.1%] men), and more likely to have tumors that were located in the upper stomach (637 patients [22.9%] vs 279 patients [55.5%] and 173 patients [43.1%]), were larger in size (median [IQR] size, 4.0 [2.5-6.0] cm vs 4.5 [3.0-6.0] cm and 4.5 [3.0-6.0] cm ), had more vascular invasion (798 patients [28.7%] vs 203 patients [49.0%] and 128 patients [34.3%]), and have more advanced disease stages (Table 1). In terms of treatment, compared with patients with gastric adenocarcinoma, the proportion of total gastrectomy in patients with gastric neuroendocrine carcinoma or gastric mixed adenoneuroendocrine carcinoma was higher (1457 patients [52.3%] vs 291 patients [57.9%] and 231 patients [57.6%]; *P* < .001), but the number of lymph nodes retrieved was lower (median [IQR], 33 [26-43] nodes vs 21 [14-30] nodes and 22 [15-31] nodes; *P* < .001). There were statistically significant differences between patients with gastric neuroendocrine carcinoma and those with gastric mixed adenoneuroendocrine carcinoma in terms of tumor location, number of lymph node metastasis, N stage, and vascular and nerve invasion, but there were no significant differences in other clinicopathological factors (Table 1).

**Table 1.** Baseline Clinicopathologic Characteristics of Included Patients

Characteristic	No. (%)			*P* value		
	AC (n = 2785)	NEC (n = 503)	MANEC (n = 401)	NEC vs AC	MANEC vs AC	NEC vs MANEC
Age, median (IQR), y^a^	61 (54-68)	64 (58-69)	64 (58-70)	<.001	<.001	.89
Sex^b^						
Men	2035 (73.1)	400 (79.5)	313 (78.1)	.002	.03	.59
Women	750 (26.9)	103 (20.5)	88 (21.9)			
Tumor location^b^						
Lower	1223 (43.9)	117 (23.3)	115 (28.7)	<.001	<.001	.002
Middle	607 (21.8)	76 (15.1)	86 (21.4)			
Upper	637 (22.9)	279 (55.5)	173 (43.1)			
Mix	209 (8.1)	31 (6.2)	27(6.7)			
Tumor size, median (IQR), cm^a^	4.0 (2.5-6.0)	4.5 (3.0-6.0)	4.5 (3.0-6.0)	<.001	<.001	.82
Lymph nodes examined, median (IQR), No.^a^	33 (26-43)	21 (14-30)	22 (15-31)	<.001	<.001	.14
Metastatic lymph nodes, median (IQR), No.^a^	2 (0-8)	2 (0-6)	3 (0-7)	.53	.006	.04
Invasion^b^^,^^c^						
Vascular	798 (28.7)	203 (49.0)	128 (34.3)	<.001	.02	<.001
Neural	761 (27.3)	145 (35.8)	103 (27.8)	<.001	.86	.02
T stage^b^						
T1	758 (27.2)	29 (5.8)	36 (9.0)	<.001	<.001	.05
T2	320 (11.5)	48 (9.5)	43 (10.7)			
T3	917 (32.9)	88 (17.5)	61 (15.2)			
T4a	760 (27.3)	328 (65.2)	260 (64.8)			
T4b	30 (1.1)	10 (2.0)	1 (0.2)			
N stage^b^						
N0	1073 (38.5)	147 (29.2)	108 (26.9)	<.001	<.001	.03
N1	447 (16.1)	113 (22.5)	85 (21.2)			
N2	490 (17.6)	139 (27.6)	98 (24.4)			
N3a	495 (17.8)	87 (17.3)	78 (19.5)			
N3b	280 (10.1)	17 (3.4)	32 (8.0)			
TNM stage^b^						
I	862 (31.0)	47 (9.3)	48 (12.0)	<.001	<.001	.38
II	659 (23.7)	144 (28.6)	105 (26.2)			
III	1264 (45.4)	312 (62.0)	248 (61.8)			
Underwent gastrectomy^b^						
Total	1457 (52.3)	291 (57.9)	231 (57.6)	.02	.047	.94
<Total	1328 (47.7)	212 (42.1)	170 (42.4)			
Received chemotherapy						
Neoadjuvant y^b^	61 (2.2)	18 (3.6)	11 (2.7)	.06	.49	.48
Adjuvant^b^^,^^d^	1645 (59.1)	283 (63.2)	226 (61.2)	.10	.42	.57

Abbreviations: AC, adenocarcinoma; IQR, interquartile range; MANEC, mixed adenoneuroendocrine carcinoma; NEC, neuroendocrine carcinoma.

^a^ Compared by using Mann-Whitney *U* tests.

^b^ Compared by using the χ^2^ test.

^c^ Vascular invasion data were missing for 117 patients, and neural invasion data were missing for 128 patients.

^d^ Adjuvant chemotherapy of 87 patients was unknown.

The median (range) follow-up time was 58.0 (1.0-153.0) months for patients with gastric neuroendocrine carcinoma, 56 (1.2-152.0) for patients with gastric mixed adenoneuroendocrine carcinoma, and 70.0 (1.5-121.0) months for patients with gastric adenocarcinoma. The incidence of recurrence was 239 patients with gastric neuroendocrine carcinoma (47.5%) and 184 patients with gastric mixed adenoneuroendocrine carcinoma (45.9%), which were significantly higher than the incidence of recurrence in patients with gastric adenocarcinoma (793 patients [28.5%]; *P* < .001) (eFigure 2 in the Supplement). Survival curves showed that the DFS and OS of patients with gastric neuroendocrine carcinoma or gastric mixed adenoneuroendocrine carcinoma were significantly worse than those of patients with gastric adenocarcinoma, while the PRSs of patients with gastric neuroendocrine carcinoma and patients with gastric mixed adenoneuroendocrine carcinoma were similar to that of patients with gastric adenocarcinoma patients (eFigure 3 in the Supplement). The recurrence pattern of gastric neuroendocrine carcinoma was similar to that of gastric mixed adenoneuroendocrine carcinoma, and both were more prone to distant recurrence than gastric adenocarcinoma (eFigure 4 in the Supplement).

### Gastric Neuroendocrine Carcinoma vs Gastric Adenocarcinoma in the Matched Data

After propensity score matching, matched data sets were developed for 500 patients with gastric neuroendocrine carcinoma and 1573 patients with gastric adenocarcinoma. There were no significant differences in stage, age, or sex between patients with gastric neuroendocrine carcinoma and those with gastric adenocarcinoma (eTable 2 in the Supplement).

#### Survival

In the matched data, the incidence of recurrence in gastric neuroendocrine carcinoma was significantly higher than that in gastric adenocarcinoma (237 patients [47.4%] vs 568 patients [36.1%]; *P* < .001) (eFigure 2 in the Supplement). Among patients with gastric neuroendocrine carcinoma, 5-year DFS was 47.6% (95% CI, 42.7%-52.5%), and 5-year OS was 50.5% (95% CI, 45.6%-55.4%); and for patients with gastric adenocarcinoma, 5-DFS was 57.6% (95% CI, 55.1%-60.1%; *P* < .001), and 5-year OS was 60.5% (95% CI, 58.0%-63.0%; *P* <  .001) (Figure, A and B). In the univariable analyses, gastric neuroendocrine carcinoma (vs gastric adenocarcinoma) was associated with reduced DFS (hazard ratio [HR], 1.36; 95% CI, 1.17-1.57; *P* < .001) (Table 2). Multivariable analysis confirmed that gastric neuroendocrine carcinoma (vs gastric adenocarcinoma) was an independent risk factor associated with worse DFS (HR, 1.64; 95% CI, 1.40-1.93; *P* < .001) (Table 2). Even compared with poorly differentiated gastric adenocarcinoma, gastric neuroendocrine carcinoma was significantly associated with worse DFS (HR, 1.64; 95%CI, 1.38-1.96; *P* < .001) (Table 2). The median (IQR) PRS of propensity score–matched patients with gastric neuroendocrine carcinoma was 8 (4-15) months, compared with 6 (3-14) months among those with gastric adenocarcinoma (*P* = .08) (eFigure 5 in the Supplement).

**Figure.**
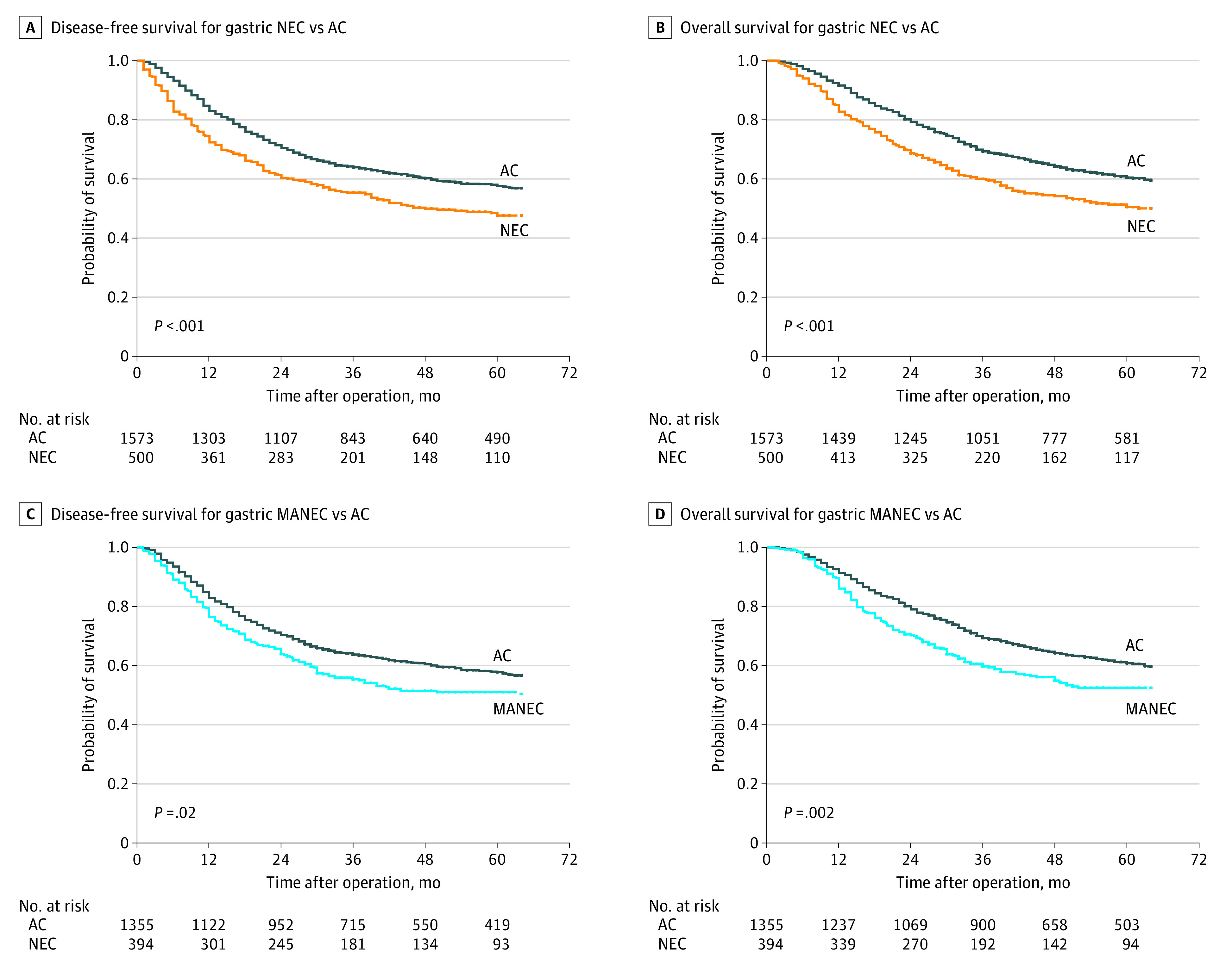
Kaplan-Meier Survival Curves for Patients With Different Histologic Subtypes of Gastric Carcinoma in the Matched Data AC indicates adenocarcinoma; MANEC, mixed adenoneuroendocrine carcinoma; and NEC, neuroendocrine carcinoma.

**Table 2.** Univariable and Multivariable Cox Regression Analyses of Factors Associated with Disease-Free Survival in the Matched Data Sets of Patients With AC vs Patients With NEC

Clinicopathological features	Univariable analysis		Multivariable analysis I^a^		Multivariable analysis II^b^	
	HR (95% CI)	*P* value	HR (95% CI)^c^	*P* value	HR (95% CI)^c^	*P* value
Age, per 1-y increase	1.02 (1.01-1.03)	<.001	1.02 (1.01-1.03)	<.001	1.02 (1.01-1.03)	<.001
Sex						
Men	1 [Reference]	.03	NA	.36	NA	.36
Women	0.83 (0.71-0.99)		NA		NA	
Center volume						
Low	1 [Reference]	.26	NA	NA	NA	NA
High	0.77 (0.49-1.21)		NA	NA	NA	NA
Tumor location						
Lower	1 [Reference]	NA	NA	NA	NA	NA
Middle	1.10 (0.91-1.33)	.32	NA	.14	NA	.14
Upper	1.21 (1.04-1.42)	.02	NA	.50	NA	.50
Mix	1.59 (1.29-1.96)	<.001	NA	.45	NA	.45
Tumor size, per 1-cm increase	1.16 (1.14-1.19)	<.001	1.06 (1.03-1.09)	<.001	1.06 (1.03-1.09)	<.001
Lymph nodes examined, per 1-unit increase	1.00 (0.99-1.00)	.25	NA	NA	NA	NA
No. of metastatic lymph nodes, per 1-unit increase	1.06 (1.06-1.07)	<.001	1.05 (1.04-1.06)	<.001	1.05 (1.04-1.06)	<.001
Invasion						
Vascular	1.72 (1.50-1.96)	<.001	NA	.93	NA	.93
Neural	1.72 (1.51-1.97)	<.001	1.24 (1.08-1.43)	.002	1.24 (1.08-1.43)	.002
T stage						
T1-T2	1 [Reference]	<.001	1 [Reference]	<.001	1 [Reference]	<.001
T3-T4	3.84 (3.02-4.90)		2.00 (1.54-2.59)		2.00 (1.54-2.59)	
N stage						
N0	1 [Reference]	<.001	1 [Reference]	<.001	1 [Reference]	<.001
N1-N3	2.91 (2.40-3.54)		1.51 (1.22-1.86)		1.51 (1.22-1.86)	
Type of gastrectomy						
Total	1 [Reference]	<.001	NA	.61	NA	.61
<Total	0.70 (0.61-0.80)		NA		NA	
Received neoadjuvant chemotherapy						
No	1 [Reference]	<.001	1 [Reference]	.001	1 [Reference]	.001
Yes	1.92 (1.36-2.72)		1.78 (1.26-2.53)		1.78 (1.26-2.53)	
Received adjuvant chemotherapy						
No	1 [Reference]	NA	NA	NA	NA	NA
Yes	1.12 (0.97-1.29)	.13	NA	NA	NA	NA
Unknown	0.96 (0.63-1.47)	.86	NA	NA	NA	NA
Pathological type I						
AC	1 [Reference]	<.001	1 [Reference]	<.001	NA	NA
NEC	1.36 (1.17-1.57)		1.64 (1.40-1.93)		NA	NA
Pathological type II						
Poorly differentiated AC	1 [Reference]	NA	NA	NA	1 [Reference]	NA
Well or moderately differentiated AC	0.72 (0.62-0.84)	<.001	NA	NA	1.00 (0.85-1.17)	.99
NEC	1.18 (1.01-1.38)	.04	NA	NA	1.64 (1.38-1.96)	<.001

Abbreviations: AC, adenocarcinoma; HR, hazard ratio: NA, not applicable; NEC, neuroendocrine carcinoma.

^a^ Multivariable analysis I included pathological type I and excluded pathological type II.

^b^ Multivariable analysis II included pathological type II, excluding pathological type I.

^c^ Because multivariate analyses used a forward likelihood ratio method, there are no corresponding HR data for factors with *P* > .05.

Stratified analysis by center volumes showed that in high-volume centers, patients with gastric neuroendocrine carcinoma had lower DFS and OS than patients with gastric adenocarcinoma, while the PRS of patients with gastric neuroendocrine carcinoma was similar to that of patients with gastric adenocarcinoma (eFigure 6 in the Supplement). Owing to the small number of patients in low-volume centers, we could not further analyze and compare the prognosis between groups.

#### Patterns of Recurrence

In the matched data, 805 patients experienced recurrent disease, of whom 715 (88.8%) had a known site of recurrence. Most patients had initial recurrence involving only a single site (gastric neuroendocrine carcinoma: 143 patients [87.8%]; gastric adenocarcinoma: 446 patients [80.9%]), and 20 patients with gastric neuroendocrine carcinoma (12.2%) and 105 patients with gastric adenocarcinoma (19.1%) had multiple recurrence (eFigure 7 in the Supplement). The detailed sites of recurrence are shown in Table 3. The incidence of distant recurrence in gastric neuroendocrine carcinoma was significantly higher than that in gastric adenocarcinoma (101 patients [23.7%] vs 268 patients [17.2%]; *P* = .002), especially in liver metastasis (61 patients [14.3%] vs 97 patients [6.2%]; *P* < .001) (Table 3). However, there was no significant difference between patients with gastric neuroendocrine carcinoma vs those with gastric adenocarcinoma in terms of locoregional recurrence, peritoneal recurrence, or multiple recurrence (Table 3). Multivariable analysis revealed that gastric neuroendocrine carcinoma (vs gastric adenocarcinoma) was an independent risk factor associated with distant recurrence (odds ratio [OR], 2.22; 95% CI, 1.66-2.98) (eTable 3 in the Supplement).

**Table 3.** Specific Sites of Recurrence in the Matched Data Sets

Variable	AC vs NEC			AC vs MANEC		
	AC (n = 1556)	NEC (n = 427)	*P* value^a^	AC (n = 1340)	MANEC (n = 333)	*P* value^a^
Locoregional recurrence, No. (%)	88 (5.7)	22 (5.2)	.69	74 (5.5)	12 (3.6)	.16
Anastomosis	43 (2.8)	16 (3.7)	.29	34 (2.5)	6 (1.8)	.43
Regional node	31 (2.0)	4 (0.9)	.14	31 (2.3)	2 (0.6)	.07
Gastric or tumor bed	22 (1.4)	2 (0.5)	.11	16 (1.2)	4 (1.2)	>.99
Recurrence, No. (%)						
Peritoneal	90 (5.8)	20 (4.7)	.38	83 (6.2)	16 (4.8)	.34
Distant	268 (17.2)	101(23.7)	.002	232 (17.3)	76 (22.8)	.02
Distant lymph node	120 (7.7)	21 (4.9)	.05	105 (7.8)	32 (9.6)	.29
Liver	97 (6.2)	61 (14.3)	<.001	81 (6.0)	42 (12.6)	<.001
Lung	44 (2.8)	13 (3.0)	.81	42 (3.1)	5 (1.5)	.11
Bone	39 (2.5)	8 (1.9)	.45	29 (2.2)	4 (1.2)	.26
Brain	6 (0.4)	2 (0.5)	>.99	3 (0.2)	2 (0.6)	.57
Pancreas	16 (1.0)	3 (0.7)	.74	12 (0.9)	4 (1.2)	.84
Spleen	7 (0.4)	0	.36	8 (0.6)	0	.37
Adrenal	11 (0.7)	3 (0.7)	>.99	8 (0.6)	0	.37
Colorectum	9 (0.6)	0	.22	5 (0.4)	0	.59
Mediastinum and pleura	12 (0.8)	0	.14	10 (0.7)	0	.23
Bile duct	5 (0.3)	0	.59	4 (0.3)	0	>.99
Subcutaneous	9 (0.6)	2 (0.5)	>.99	8 (0.6)	0	.37
Other^b^	7 (0.4)	0	.36	7 (0.5)	0	.36
Multiple, No. (%)	105 (6.7)	20 (4.7)	.12	85 (6.3)	15 (4.5)	.21

Abbreviations: AC, adenocarcinoma; MANEC, mixed adenoneuroendocrine carcinoma; NEC, neuroendocrine carcinoma.

^a^ Calculated by using χ^2^ test or Fisher exact test.

^b^ Other sites included jejunum, ureter, esophagus, testis, muscle, and wound.

### Gastric Mixed Adenoneuroendocrine Carcinoma vs Gastric Adenocarcinoma in the Matched Data

After propensity score matching, 394 patients with gastric mixed adenoneuroendocrine carcinoma and 1355 patients with gastric mixed adenoneuroendocrine carcinoma formed the matched data sets. There were no significant differences in stage, age, or sex between patients with gastric mixed adenoneuroendocrine carcinoma vs those with gastric adenocarcinoma (eTable 2 in the Supplement).

#### Survival

In the matched data, the incidence of recurrence was 180 patients with gastric mixed adenoneuroendocrine carcinoma (45.7%) and 489 patients with gastric adenocarcinoma (36.1%) (*P* < .001) (eFigure 2 in the Supplement). Kaplan-Meier survival curves are shown in the Figure. The DFS and OS of patients with gastric neuroendocrine carcinoma were significantly worse than those of patients with gastric mixed adenoneuroendocrine carcinoma (5-year DFS: 51.1% [95% CI, 46.0%-56.2%] vs 57.8% [95% CI, 55.1%-60.5%]; *P* = .02; 5-year OS: 52.5% [95% CI, 47.2%-57.8%] vs 60.8% [95% CI, 57.3%-64.3%]; *P* = .002) (Figure, C and D). However, there was no significant difference in median PRS between the matched gastric mixed adenoneuroendocrine carcinoma group and gastric adenocarcinoma group (eFigure 5 in the Supplement). In the univariable analyses, gastric mixed adenoneuroendocrine carcinoma (vs gastric adenocarcinoma) was significantly associated with reduced DFS (Table 4). Multivariable analysis confirmed that gastric neuroendocrine carcinoma (vs gastric adenocarcinoma) was an unfavorable risk factor associated with worse DFS (HR, 1.25; 95%CI, 1.05-1.49) (Table 4). Similarly, gastric mixed adenoneuroendocrine carcinoma was still associated with worse DFS compared with poorly differentiated gastric adenocarcinoma (HR, 1.27; 95% CI, 1.06-1.54) (Table 4).

**Table 4.** Univariable and Multivariable Cox Regression Analyses of Factors Associated With Disease-Free Survival in the Matched Data Sets of Patients With AC vs Patients With MANEC

Clinicopathological features	Analysis					
	Univariable		Multivariable I^a^		Multivariable II^b^	
	HR (95% CI)	*P* value	HR (95% CI)^c^	*P* value	HR (95% CI)^c^	*P* value
Age	1.02 (1.01-1.03)	<.001	1.02 (1.01-1.03)	<.001	1.02 (1.01-1.03)	<.001
Sex						
Men	1 [Reference]	.31	NA	NA	NA	NA
Women	0.91 (0.76-1.09)		NA	NA	NA	NA
Center volume						
Low	1 [Reference]	.049	NA	.27	NA	.26
High	0.56 (0.32-1.00)		NA		NA	
Location						
Lower	1 [Reference]	NA	NA	NA	NA	NA
Middle	1.06 (0.87-1.30)	.58	NA	.12	NA	.11
Upper	1.24 (1.04-1.47)	.02	NA	.45	NA	.43
Mix	1.70 (1.36-2.12)	<.001	NA	.31	NA	.49
Tumor size, per 1-cm increase	1.15 (1.12-1.18)	<.001	NA	.05	1.03 (1.00-1.06)	.05
Lymph nodes examined, per 1-cm increase	1.00 (1.00-1.01)	.62	NA	NA	NA	NA
No. of metastatic lymph nodes, per 1-unit increase	1.06 (1.06-1.07)	<.001	1.05 (1.04-1.06)	<.001	1.05 (1.04-1.06)	<.001
Invasion						
Vascular	1.74 (1.51-2.01)	<.001	NA	.51	NA	.52
Neural	1.83 (1.58-2.12)	<.001	1.26 (1.09-1.47)	.002	1.28 (1.10-1.49)	.001
T stage						
T1-T2	1 [Reference]	<.001	1 [Reference]	<.001	1 [Reference]	<.001
T3-T4	4.47 (3.43-5.83)		2.62 (1.98-3.48)		2.48 (1.86-3.31)	
N stage						
N0	1 [Reference]	<.001	1 [Reference]	<.001	1 [Reference]	<.001
N1-N3	3.33 (2.67-4.16)		1.58 (1.24-2.01)		1.56 (1.23-1.99)	
Type of gastrectomy						
Total	1 [Reference]	<.001	NA	.54	NA	.32
Subtotal	0.75 (0.65-0.87)		NA		NA	
Received neoadjuvant chemotherapy						
No	1 [Reference]	.003	1 [Reference]	.003	NA	NA
Yes	1.90 (1.25-2.87)		1.86 (1.23-2.83)		NA	NA
Received adjuvant chemotherapy						
No	1 [Reference]	NA	NA	NA	NA	NA
Yes	1.117 (0.968-1.286)	.13	NA	NA	NA	NA
Unknown	0.987 (0.529-1.244)	.89	NA	NA	NA	NA
Pathological type I						
AC	1 [Reference]	.02	1 [Reference]	.01	NA	NA
MANEC	1.21 (1.03-1.43)		1.25 (1.05-1.49)		NA	NA
Pathological type II						
Poorly differentiated AC	1 [Reference]	NA	NA	NA	1 [Reference]	NA
Well or moderately differentiated AC	0.73 (0.62-0.86)	<.001	NA	NA	1.07 (0.90-1.27)	.47
MANEC	1.05 (0.88-1.25)	.59	NA	NA	1.27 (1.06-1.54)	.01

Abbreviations: AC, adenocarcinoma; HR, hazard ratio; MANEC, mixed adenoneuroendocrine carcinoma; NA, not applicable.

^a^ Multivariate Analysis I included pathological type I, excluding pathological type II.

^b^ Multivariate Analysis II included pathological type II, excluding pathological type I.

^c^ Because multivariate analyses used a forward likelihood ratio method, there are no corresponding HR data for factors with *P* > .05.

Stratified analysis confirmed that the DFS and OS of patients with gastric mixed adenoneuroendocrine carcinoma were significantly worse than those of patients with gastric adenocarcinoma in the high-volume centers. The PRS of patients with gastric mixed adenoneuroendocrine carcinoma was similar to that of patients with gastric adenocarcinoma (eFigure 8 in the Supplement).

#### Patterns of Recurrence

A total of 669 patients experienced recurrent disease in the matched data, of whom 593 (88.6%) had a known site of recurrence. The rate of recurrence involving only a single area was 104 patients with gastric mixed adenoneuroendocrine carcinoma (87.3%) and 389 patients with gastric adenocarcinoma (82.0%) (eFigure 7 in the Supplement). The incidence of distant recurrence in patients with gastric mixed adenoneuroendocrine carcinoma was significantly higher than that in patients with gastric adenocarcinoma (76 patients [22.8%] vs 232 patients [17.3%]; *P* = .02), especially in liver metastasis (42 patients [12.6%] vs 81 patients [6.0%]; *P* < .001) (Table 3). However, there was no significant difference between gastric mixed adenoneuroendocrine carcinoma and gastric adenocarcinoma in other patterns of recurrence (Table 3). Multivariable analysis confirmed that gastric mixed adenoneuroendocrine carcinoma (vs gastric adenocarcinoma) was an independent risk factor associated with distant recurrence (OR, 1.70; 95% CI, 1.24-2.34) (eTable 4 in the Supplement).

### Pathological Factors Associated With Distant Recurrence of Gastric Neuroendocrine Carcinoma and Gastric Mixed Adenoneuroendocrine Carcinoma

In this study, 178 patients with gastric neuroendocrine carcinoma or gastric mixed adenoneuroendocrine carcinoma experienced distant recurrence. In univariable analyses, T3-T4 stage, lymph node metastasis, Ki-67 expression greater than 20%, and tumors located in the middle of the stomach were associated with increased risk of distant recurrence (eTable 5 in the Supplement). However, compared with gastric neuroendocrine carcinoma, gastric mixed adenoneuroendocrine carcinoma was not associated with increased distant recurrence (eTable 5 in the Supplement). In multivariable analyses, only T3-T4 stage (OR, 2.84; 95% CI, 1.57-5.14; *P* = .001) and lymph node metastasis (OR, 2.01; 95% CI, 1.31-3.10; *P* = .002) were independent risk factors associated with distant recurrence of gastric neuroendocrine carcinoma and gastric mixed adenoneuroendocrine carcinoma (eTable 5 in the Supplement).

## Discussion

To our knowledge, this cohort study, including 3689 patients in 23 centers in China, is the first and largest multicenter retrospective study comparing the outcomes and patterns of recurrence in gastric neuroendocrine carcinoma, gastric mixed adenoneuroendocrine carcinoma, and gastric adenocarcinoma. This study found that patients with gastric neuroendocrine carcinoma or gastric mixed adenoneuroendocrine carcinoma had worse prognoses than those with gastric adenocarcinoma and that gastric neuroendocrine carcinoma and gastric mixed adenoneuroendocrine carcinoma were independent risk factors associated with worse DFS and PRS. In addition, patients with gastric neuroendocrine carcinoma or gastric mixed adenoneuroendocrine carcinoma were more likely to experience distant recurrence. Our results could provide important medical evidence for the development of follow-up and treatment strategies for patients with gastric neuroendocrine carcinoma or gastric mixed adenoneuroendocrine carcinoma.

Owing to the rarity of gastric neuroendocrine carcinoma and gastric mixed adenoneuroendocrine carcinoma, large reports with comparisons of prognosis among these patients and those with gastric adenocarcinoma are lacking. A 2017 single-center study in South Korea^6^ found that patients with gastric neuroendocrine carcinoma had worse survival than those with conventional gastric cancer, but there were significant differences in stage between patients with gastric neuroendocrine carcinoma and those with conventional gastric cancer. In our study, patients with gastric neuroendocrine carcinoma or gastric mixed adenoneuroendocrine carcinoma also had later stages and worse prognoses than those with gastric adenocarcinoma, which was consistent with previous studies.^5,6,7^ Thus, propensity score matching was used to balance the differences in stage. In the matched data sets, we found that the DFS and OS of patients with gastric neuroendocrine carcinoma or gastric mixed adenoneuroendocrine carcinoma were also worse than those of patients with gastric adenocarcinoma. Nearly half of patients with gastric neuroendocrine carcinoma or gastric mixed adenoneuroendocrine carcinoma experienced recurrence, but the prognosis of patients with recurrent gastric neuroendocrine carcinoma or gastric mixed adenoneuroendocrine carcinoma is still unknown. Therefore, more active and effective multidisciplinary treatments should be undertaken for patients with gastric neuroendocrine carcinoma or gastric mixed adenoneuroendocrine carcinoma after surgery to improve the survival time.

Poorly differentiated adenocarcinoma is a pathological type of gastric cancer associated with worse prognosis.^30,31^ To our knowledge, only 1 single-center study^6^ has reported that the recurrence-free survival rate of patients with poorly differentiated gastric adenocarcinoma was lower than that of patients with gastric neuroendocrine carcinoma, but reports comparing survival between patients with poorly differentiated gastric adenocarcinoma and those with gastric mixed adenoneuroendocrine carcinoma are still lacking. In our study, after adjusting for a number of factors associated with worse prognosis, multivariable analysis found that gastric neuroendocrine carcinoma and gastric mixed adenoneuroendocrine carcinoma were associated with a worse prognosis than poorly differentiated gastric adenocarcinoma, suggesting that gastric neuroendocrine carcinoma and gastric mixed adenoneuroendocrine carcinoma are more malignant pathological types than poorly differentiated gastric adenocarcinoma.

The analysis and understanding of tumor recurrence patterns are conducive to optimizing follow-up and treatment strategies.^9^ However, to our knowledge, there have been no previous studies on the recurrence patterns of gastric neuroendocrine carcinoma and gastric mixed adenoneuroendocrine carcinoma. In this study, the rates of locoregional recurrence and peritoneal recurrence of gastric neuroendocrine carcinoma and gastric mixed adenoneuroendocrine carcinoma were similar to those of gastric adenocarcinoma. However, nearly three-fourths of patients with recurrent gastric neuroendocrine carcinoma or gastric mixed adenoneuroendocrine carcinoma experienced distant recurrence, and the incidence of recurrence was significantly higher for these patients than that in patients with gastric adenocarcinoma. The difference in distant recurrence mainly concerned liver metastasis. In the multivariate model, we further found that gastric neuroendocrine carcinoma and gastric mixed adenoneuroendocrine carcinoma were independent risk factors associated with distant recurrence. For gastric neuroendocrine carcinoma and gastric mixed adenoneuroendocrine carcinoma, patients with T3 to T4 stage and lymph node metastasis were at high risk of postoperative distant recurrence. Thus, in future follow-up work, appropriate examinations should be performed for patients with high-risk gastric neuroendocrine carcinoma or gastric mixed adenoneuroendocrine carcinoma, such as contrast-enhanced computed tomography or magnetic resonance imaging, to improve the detection rate of distant recurrence. Adjuvant therapies focusing on distant recurrence should be used and developed to improve the long-term survival of patients with gastric neuroendocrine carcinoma or gastric mixed adenoneuroendocrine carcinoma.

### Limitations

This study has several limitations. First, patients with gastric adenocarcinoma were only included from a single center, which may have caused selection bias. Thus, propensity score matching was used to reduce the potential interference associated with stage difference. Second, this study was performed in China, so it is not clear whether our findings are generalizable for Western populations. Third, nearly 10.0% of patients with recurrence did not have a documented recurrence pattern, and these patients had to be excluded from the recurrence pattern analysis, which may have led to selection bias. Fourth, patients with gastric adenocarcinoma patients were only included from one center, which may have produced center-related effects. Thus, stratified analyses by center volume were performed, and we found that even in the high-volume centers, the long-term survival of patients with gastric neuroendocrine carcinoma or gastric mixed adenoneuroendocrine carcinoma was significantly worse than that of patients with gastric adenocarcinoma. Nevertheless, to our knowledge, this study represents the largest multicenter study focusing on recurrence patterns of gastric neuroendocrine carcinoma and gastric mixed adenoneuroendocrine carcinoma, which could support the development of guidelines and suggests the need for prospective studies.

## Conclusions

This cohort study found that gastric neuroendocrine carcinoma and gastric mixed adenoneuroendocrine carcinoma were associated with a worse prognosis than gastric adenocarcinoma and even poorly differentiated gastric adenocarcinoma. In addition, patients with gastric neuroendocrine carcinoma and gastric mixed adenoneuroendocrine carcinoma were more likely to experience distant recurrence, especially patients with tumors penetrating into the subserosa or deeper layers and with lymph node metastasis. Thus, additional close follow-up strategies and effective adjuvant therapies, such as chemotherapy combined with targeted therapy or immunotherapy, should be implemented for these patients.

## Notes

Supplement. **eTable 1.** Distribution of Multicenter Patients **eTable 2.** Comparison of Stages Between Different Pathological Types in the Original and the Matched Data Sets **eTable 3.** Univariable and Multivariable Logistic Regression Analyses of Factors Potentially Associated with Distant Recurrence in the Matched Data Sets of AC and NEC **eTable 4.** Univariate and Multivariable Logistic Regression Analyses of Factors Potentially Associated with Distant Recurrence in the Matched Data Sets of AC and MANEC **eTable 5.** Univariable and Multivariable Logistic Regression Analyses of Pathological Factors Potentially Associated with Distant Recurrence in Patients with NEC and MANEC **eFigure 1.** Diagram Representing the Selection of the Study Population **eFigure 2.** Incidence of Recurrence According to Histologic Subtype in Original Data and Matched Data **eFigure 3.** Kaplan-Meier Survival Curves for Patients With Gastric Neuroendocrine Carcinoma (NEC), Mixed Adenoneuroendocrine Carcinoma (MANEC), or Adenocarcinoma (AC) in Original Data **eFigure 4.** Recurrence Patterns According to Histologic Subtype in the Original Data **eFigure 5.** Kaplan-Meier Survival Curves for Postrecurrence Survival for Patients With Different Histologic Subtypes in the Matched Data **eFigure 6.** Kaplan-Meier Survival Curves for Patients With Gastric Neuroendocrine Carcinoma (NEC) and Adenocarcinoma (AC) in High-Volume Centers **eFigure 7.** Recurrence Patterns According to Histologic Subtype in the Matched Data **eFigure 8.** Kaplan-Meier Survival Curves for Patients With Gastric Mixed Adenoneuroendocrine Carcinoma (MANEC) and Adenocarcinoma (AC) in High-Volume Centers
