# Supplementary material for: Comparison of Survival and Patterns of Recurrence in Gastric Neuroendocrine Carcinoma, Mixed Adenoneuroendocrine Carcinoma, and Adenocarcinoma
Source: JAMA Netw Open. 2021 Jul 27;4(7):e2114180. doi: 10.1001/jamanetworkopen.2021.14180 (PMC8317013; doi:10.1001/jamanetworkopen.2021.14180)
Supplement: Supplement. — eTable 1. Distribution of Multicenter Patients eTable 2. Comparison of Stages Between Different Pathological Types in the Original and the Matched Data Sets eTable 3. Univariable and Multivariable Logistic Regression Analyses of Factors Potentially Associated with Distant Recurrence in the Matched Data Sets of AC and NEC eTable 4. Univariate and Multivariable Logistic Regression Analyses of Factors Potentially Associated with Distant Recurrence in the Matched Data Sets of AC and MANEC eTable 5. Univariable and Multivariable Logistic Regression Analyses of Pathological Factors Potentially Associated with Distant Recurrence in Patients with NEC and MANEC eFigure 1. Diagram Representing the Selection of the Study Population eFigure 2. Incidence of Recurrence According to Histologic Subtype in Original Data and Matched Data eFigure 3. Kaplan-Meier Survival Curves for Patients With Gastric Neuroendocrine Carcinoma (NEC), Mixed Adenoneuroendocrine Carcinoma (MANEC), or Adenocarcinoma (AC) in Original Data eFigure 4. Recurrence Patterns According to Histologic Subtype in the Original Data eFigure 5. Kaplan-Meier Survival Curves for Postrecurrence Survival for Patients With Different Histologic Subtypes in the Matched Data eFigure 6. Kaplan-Meier Survival Curves for Patients With Gastric Neuroendocrine Carcinoma (NEC) and Adenocarcinoma (AC) in High-Volume Centers eFigure 7. Recurrence Patterns According to Histologic Subtype in the Matched Data eFigure 8. Kaplan-Meier Survival Curves for Patients With Gastric Mixed Adenoneuroendocrine Carcinoma (MANEC) and Adenocarcinoma (AC) in High-Volume Centers [file jamanetwopen-e2114180-s001.pdf]

Supplemental Online Content

Lin J, Zhao Y, Zhou Y, et al. Comparison of survival and patterns of recurrence in gastric neuroendocrine carcinoma, mixed adenoneuroendocrine carcinoma, and adenocarcinoma. *JAMA Netw Open*. 2021;4(7):e2114180. doi:10.1001/jamanetworkopen.2021.14180

eTable 1. Distribution of Multicenter Patients
eTable 2. Comparison of Stages Between Different Pathological Types in the Original and the Matched Data Sets
eTable 3. Univariable and Multivariable Logistic Regression Analyses of Factors Potentially Associated with Distant Recurrence in the Matched Data Sets of AC and NEC
eTable 4. Univariate and Multivariable Logistic Regression Analyses of Factors Potentially Associated with Distant Recurrence in the Matched Data Sets of AC and MANEC
eTable 5. Univariable and Multivariable Logistic Regression Analyses of Pathological Factors Potentially Associated with Distant Recurrence in Patients with NEC and MANEC
eFigure 1. Diagram Representing the Selection of the Study Population
eFigure 2. Incidence of Recurrence According to Histologic Subtype in Original Data and Matched Data
eFigure 3. Kaplan-Meier Survival Curves for Patients With Gastric Neuroendocrine Carcinoma (NEC), Mixed Adenoneuroendocrine Carcinoma (MANEC), or Adenocarcinoma (AC) in Original Data
eFigure 4. Recurrence Patterns According to Histologic Subtype in the Original Data
eFigure 5. Kaplan-Meier Survival Curves for Postrecurrence Survival for Patients With Different Histologic Subtypes in the Matched Data
eFigure 6. Kaplan-Meier Survival Curves for Patients With Gastric Neuroendocrine Carcinoma (NEC) and Adenocarcinoma (AC) in High-Volume Centers
eFigure 7. Recurrence Patterns According to Histologic Subtype in the Matched Data
eFigure 8. Kaplan-Meier Survival Curves for Patients With Gastric Mixed Adenoneuroendocrine Carcinoma (MANEC) and Adenocarcinoma (AC) in High-Volume Centers

This supplemental material has been provided by the authors to give readers additional information about their work.

© 2021 Lin J et al. *JAMA Network Open.*

eTable 1. Distribution of Multicenter Patients

Center	number of patients
Fujian Medical University Union Hospital	155
West district of The First Affiliated Hospital of USTC	115
The Affliated Hospital of Qingdao University	84
Cancer Hospital Affiliated to Chinese Academy of Medical Sciences	83
The First Affiliated Hospital of Fujian Medical University	80
Huashan Hospital Affiliated to Fudan University	66
The First Affiliated Hospital of Anhui Medical University	53
Yantai Yuhuangding Hospital	51
Renji Hospital Affiliated to Shanghai Jiaotong University	50
The Affliated Hospital of Putian University	43
The First Hospital of Putian	36
Henan Cancer Hospital	30
Fujian Provincial Hospital	13
The Second People's Hospital of Liaocheng	7
Tianjin Medical University General Hospital	6
The First Affiliated Hospital of Nanjing Medical University	6
Guangdong Provincial People's Hospital	5
Quanzhou First Hospital Affiliated to Fujian Medical University	5
The First Affiliated Hospital of Soochow University	4
Ruijin Hospital Affiliated to Shanghai Jiao Tong University School of Medicine	4
Zhangzhou Affiliated Hospital of Fujian Medical University	3
Meizhou People's Hospital	3
Second Affiliated Hospital of Nanchang University	2

© 2021 Lin J et al. *JAMA Network Open.*

eTable 2. Comparison of Stages Between Different Pathological Types in the Original and the Matched Data Sets

Baseline Variable	Before matching			After matching		
	AC (n=2785, %)	NEC (n=503, %)	P value	AC (n=1573, %)	NEC (n=500, %)	P value
Age, median (IQR), y	61 (54-68)	64 (58-69)	<.001	63 (58-69)	64 (58-69)	.40
Sex			.002			.74
Male	2035 (73.1)	400 (79.5)		1263 (80.3)	398 (79.6)	
Female	750 (26.9)	103 (20.5)		310 (19.7)	102 (20.4)	
Pathologic TNM stage			<.001			.32
I	862 (31.0)	47 (9.3)		186 (11.8)	47 (9.4)	
II	659 (23.7)	144 (28.6)		433 (27.5)	144 (28.8)	
III	1264 (45.4)	312 (62.0)		954 (60.6)	309 (61.8)	
	AC (n=2785, %)	MANEC (n=401, %)	P value	AC (n=1355, %)	MANEC (n=394, %)	P value
Age, median (IQR), y	61 (54-68)	64 (58-70)	<.001	63 (57-69)	64 (58-70)	.09
Sex			.03			.69
Male	2035 (73.1)	313 (78.1)		1082 (79.9)	311 (78.9)	
Female	750 (26.9)	88 (21.9)		273 (20.1)	83 (21.1)	
Pathologic TNM stage			<.001			.56
I	862 (31.0)	48 (12.0)		190 (14.0)	48 (12.2)	
II	659 (23.7)	105 (26.2)		361 (26.6)	102 (25.9)	
III	1264 (45.4)	248 (61.8)		804 (59.3)	244 (61.9)	

Abbreviations: NEC, neuroendocrine carcinoma; MANEC, mixed adenoneuroendocrine carcinoma; AC, adenocarcinoma; Sdiff, standardized difference.

© 2021 Lin J et al. *JAMA Network Open.*

eTable 3. Univariable and Multivariable Logistic Regression Analyses of Factors Potentially Associated with Distant Recurrence in the Matched Data Sets of AC and NEC

Clinicopathological features	Univariable Analysis		Multivariable analysis	
	Odds Ratio (CI)	P	Odds Ratio (CI)	P
Age	1.02 (1.00-1.03)	**.02**	1.02 (1.00-1.03)	**.04**
Sex				
Male	1		1	
Female	0.63 (0.46-0.86)	**.003**	0.66 (0.47-0.93)	**.02**
Center volume				
Low	1			
High	1.04 (0.39-2.78)	.94		
Location				
Lower	1		—	
Middle	1.15 (0.82-1.61)	.42	—	.17
Upper	1.47 (1.12-1.94)	**.006**	—	.59
Mix	1.51 (1.03-2.22)	**.04**	—	.61
Tumor size	1.16 (1.11-1.22)	**<.001**	—	.59
Lymph nodes examined	1.00 (1.00-1.01)	.43		
The number of metastatic lymph nodes	1.08 (1.07-1.10)	**<.001**	1.06 (1.05-1.08)	**<.001**
Vascular invasion	1.48 (1.17-1.88)	**.001**	—	.17
Neural invasion	1.30 (1.03-1.66)	**.03**	—	.10
Pathologic T stage				
T1-2	1		1	
T3-4	4.43 (2.92-6.71)	**<.001**	2.19 (1.40-3.41)	**.001**
Pathologic N stage				
N0	1		1	
N1-3	5.43 (3.64-8.10)	**<.001**	2.94 (1.89-4.55)	**<.001**
Type of resection				
Total gastrectomy	1		1	
Subtotal gastrectomy	0.57 (0.45-0.72)	**<.001**	0.77 (0.59-1.00)	**.05**
Received neoadjuvant chemotherapy				
No	1		1	
Yes	2.95 (1.57-5.55)	**.001**	2.86 (1.44-5.67)	**.003**
Received adjuvant chemotherapy				
No	1		1	
Yes	1.46 (1.13-1.89)	**.004**	—	.91
Pathological type				
AC	1		1	
NEC	1.46 (1.12-1.89)	**.005**	2.22 (1.66-2.98)	**<.001**

Abbreviations: NEC, neuroendocrine carcinoma; AC, adenocarcinoma; CI, confidence interval.

© 2021 Lin J et al. *JAMA Network Open.*

eTable 4. Univariate and Multivariable Logistic Regression Analyses of Factors Potentially Associated with Distant Recurrence in the Matched Data Sets of AC and MANEC

Clinicopathological features	Univariable Analysis		Multivariable analysis	
	Odds Ratio (CI)	P	Odds Ratio (CI)	P
Age	1.01 (1.00-1.03)	.09		
Sex				
Male	1		1	
Female	0.67 (0.48-0.94)	**.02**	0.69 (0.48-1.00)	**.05**
Center volume				
Low	1			
High	0.81 (0.22-2.96)	.75		
Location				
Lower	1		—	
Middle	1.03 (0.73-1.47)	.86	—	.07
Upper	1.54 (1.14-2.08)	**.005**	—	.06
Mix	1.51 (0.99-2.29)	.06	—	.63
Tumor size	1.17 (1.11-1.22)	**<.001**	—	.97
Lymph nodes examined	1.00 (0.99-1.01)	.61		
The number of metastatic lymph nodes	1.09 (1.07-1.11)	**<.001**	1.07 (1.05-1.09)	**<.001**
Vascular invasion	1.62 (1.25-2.09)	**<.001**	—	.73
Neural invasion	1.48 (1.14-1.92)	**.003**	—	.43
Pathologic T stage				
T1-2	1		1	
T3-4	6.81 (4.11-11.27)	**<.001**	3.52 (2.08-5.97)	**<.001**
Pathologic N stage				
N0	1		1	
N1-3	5.12 (3.35-7.84)	**<.001**	2.08 (1.30-3.32)	**.002**
Type of resection				
Total gastrectomy	1			
Subtotal gastrectomy	0.63 (0.48-0.81)	**<.001**		
Received neoadjuvant chemotherapy				
No	1		1	
Yes	2.89 (1.41-5.92)	**.004**	2.97 (1.40-6.33)	**.005**
Received adjuvant chemotherapy				
No	1		1	
Yes	1.78 (1.34-2.36)	**<.001**	—	.48
Pathological type				
AC	1		1	
MANEC	1.39 (1.03-1.86)	**.03**	1.70 (1.24-2.34)	**.001**

Abbreviations: MANEC, mixed adenoneuroendocrine carcinoma; AC, adenocarcinoma; CI, confidence interval.

© 2021 Lin J et al. *JAMA Network Open.*

eTable 5. Univariable and Multivariable Logistic Regression Analyses of Pathological Factors Potentially Associated with Distant Recurrence in Patients with NEC and MANEC

Clinicopathological features	Univariable Analysis		Multivariable analysis	
	Odds Ratio (95%CI)	P	Odds Ratio (95%CI)	P
Location				
Lower	1		—	
Middle	**0.50 (0.29-0.86)**	**.01**	—	.08
Upper	0.77 (0.52-1.14)	.20	—	.51
Mix	0.72 (0.32-1.60)	.42	—	.87
Tumor size	1.04 (0.98-1.11)	.20		
Lymph nodes examined	1.01 (0.99-1.02)	.47		
Vascular invasion	1.16 (0.80-1.66)	.44		
Neural invasion	0.90 (0.60-1.33)	.58		
Ki-67 (%)				
≤20	1		—	
>20	**2.37 (1.05-5.37)**	**.04**	—	.11
Unknown	0.78 (0.28-2.18)	.63	—	.47
Pathological type				
NEC	1			
MANEC	0.96 (0.68-1.34)	.79		
Pathologic T stage				
T1-2	1		1	
T3-4	**3.47 (1.94-6.19)**	**<.001**	**2.84 (1.57-5.14)**	**.001**
Pathologic N stage				
N0	1		1	
N1-3	**2.40 (1.57-3.65)**	**<.001**	**2.01 (1.31-3.10)**	**.002**

Abbreviations: NEC, neuroendocrine carcinoma; MANEC, mixed adenoneuroendocrine carcinoma; CI, confidence interval.

© 2021 Lin J et al. *JAMA Network Open.*

eFigure 1. Diagram Representing the Selection of the Study Population

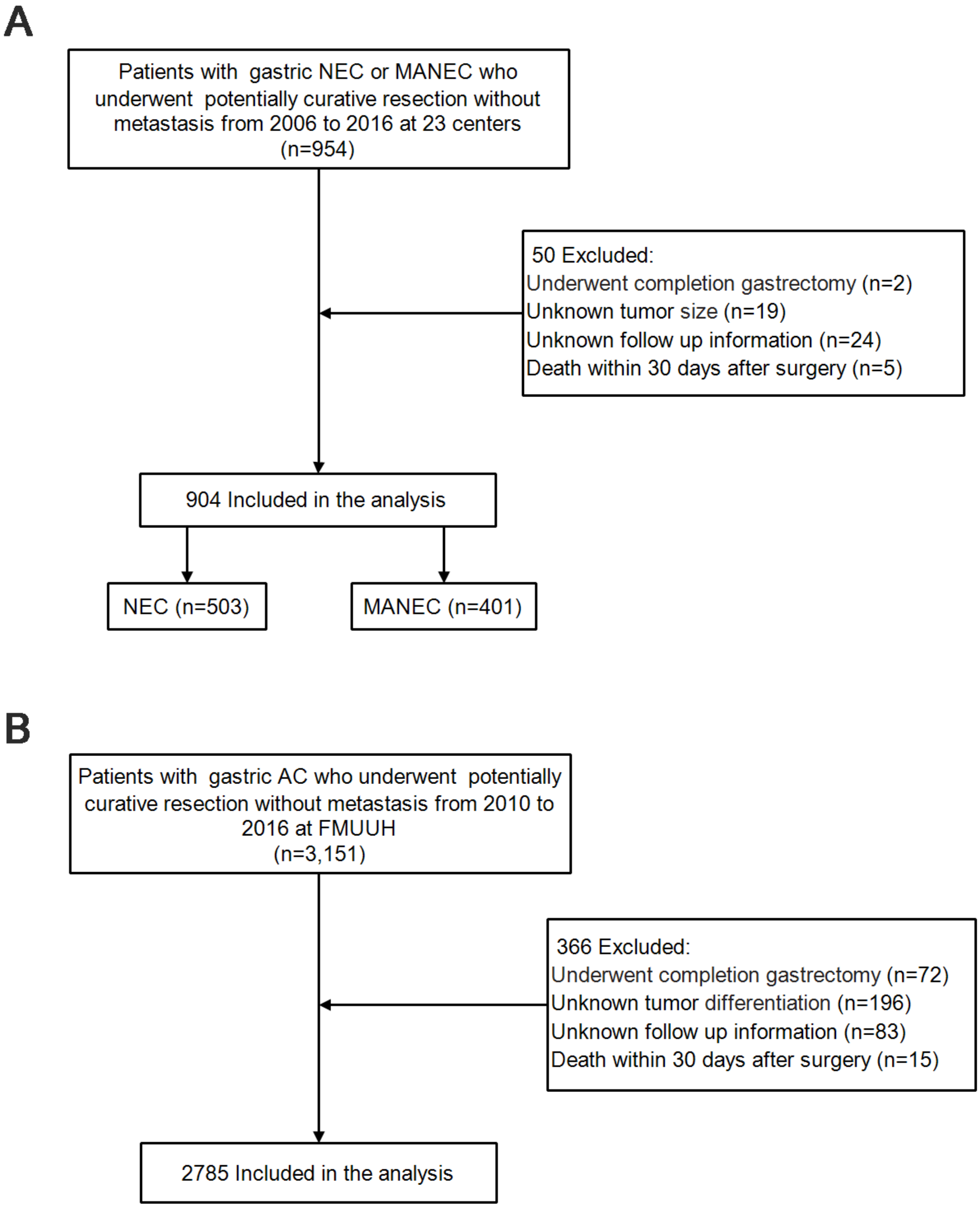

(A) Patients with gastric neuroendocrine carcinoma (NEC) and mixed adenoneuroendocrine carcinoma (MANEC). (B) Patients with gastric adenocarcinoma (AC). Abbreviations: FMUUH, Fujian Medical University Union Hospital.

© 2021 Lin J et al. *JAMA Network Open.*

eFigure 2. Incidence of Recurrence According to Histologic Subtype in Original Data and Matched Data

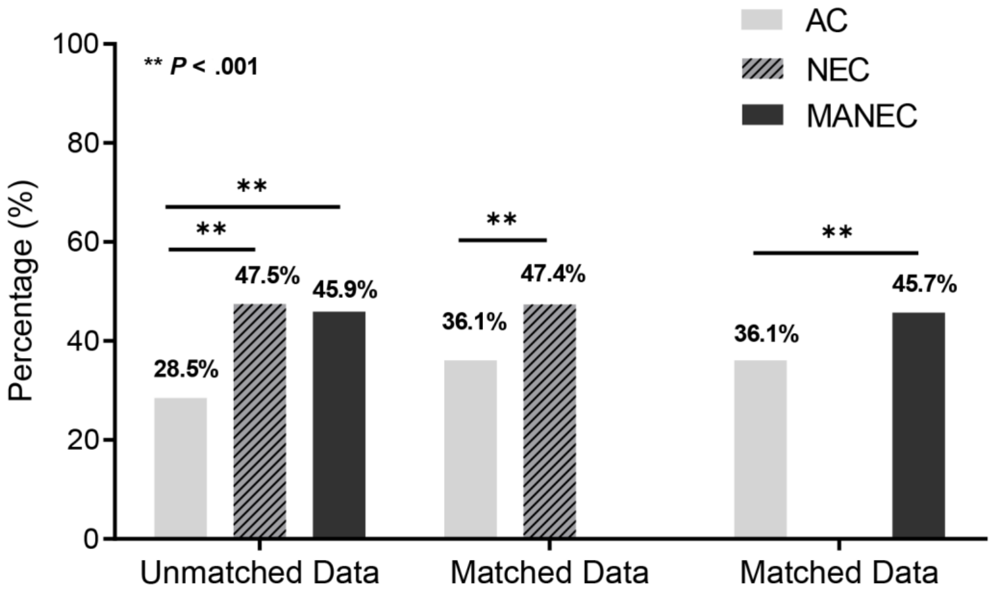

Abbreviations: NEC, neuroendocrine carcinoma. MANEC, mixed adenoneuroendocrine carcinoma. AC, adenocarcinoma

© 2021 Lin J et al. *JAMA Network Open.*

eFigure 3. Kaplan-Meier Survival Curves for Patients With Gastric Neuroendocrine Carcinoma (NEC), Mixed Adenoneuroendocrine Carcinoma (MANEC), or Adenocarcinoma (AC) in Original Data

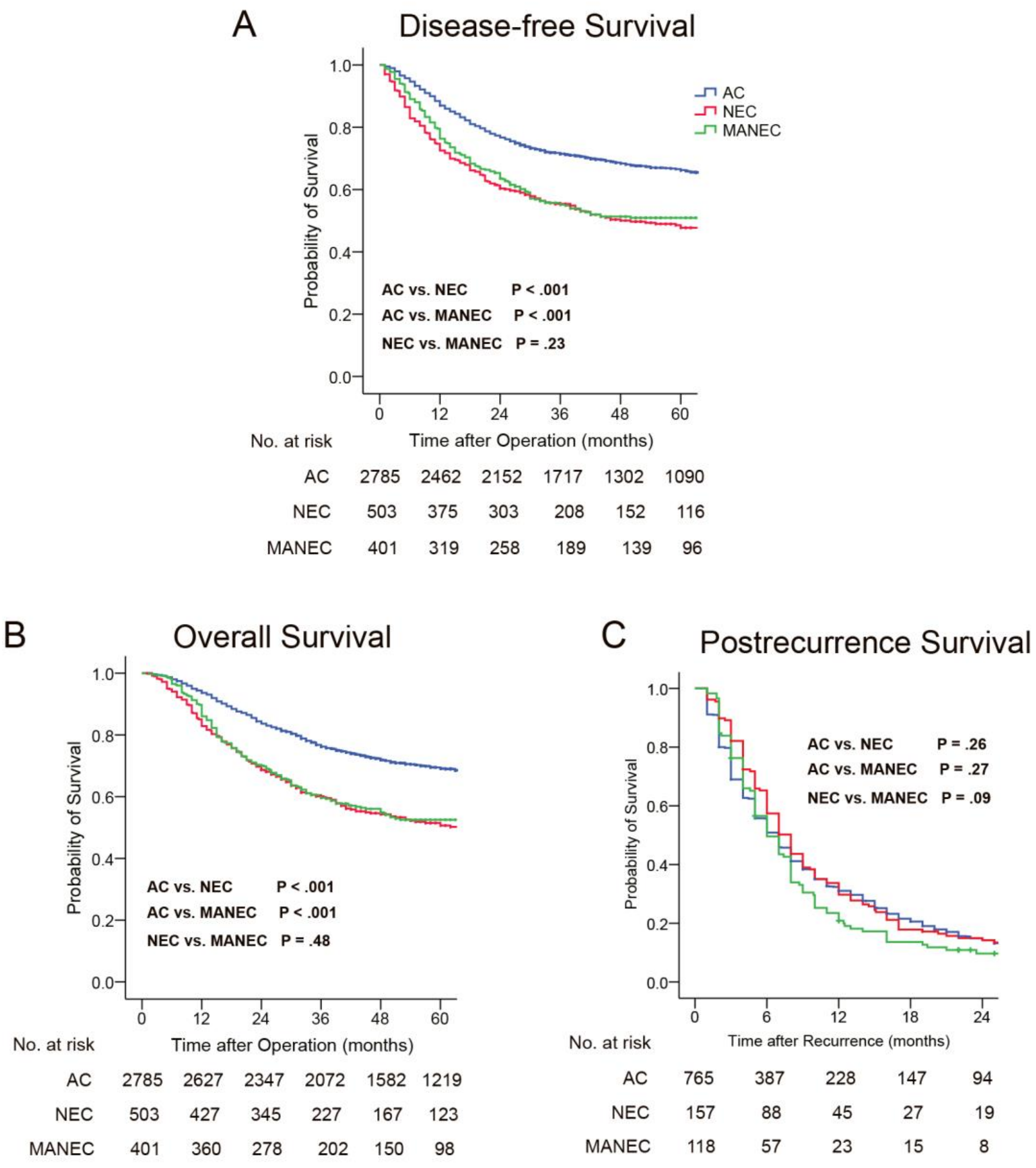

(A) Disease-free survival (DFS), (B) Overall survival (OS), and (C) postrecurrence survival (PRS).

© 2021 Lin J et al. *JAMA Network Open.*

eFigure 4. Recurrence Patterns According to Histologic Subtype in the Original Data

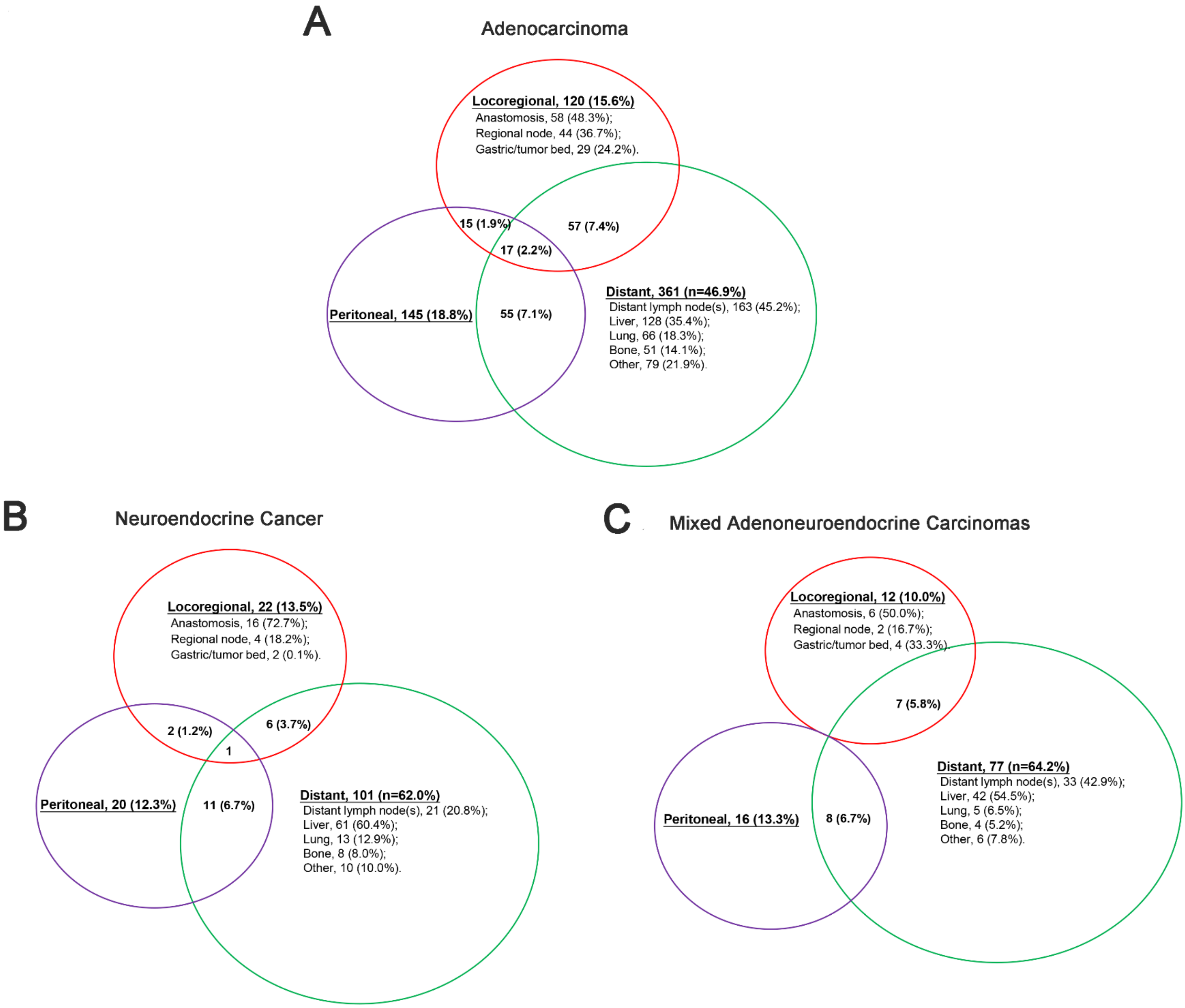

(A) adenocarcinoma, (B) neuroendocrine carcinoma, and (C) mixed adenoneuroendocrine carcinoma. Sizes of circles are proportional to the number of patients.

© 2021 Lin J et al. *JAMA Network Open.*

eFigure 5. Kaplan-Meier Survival Curves for Postrecurrence Survival for Patients With Different Histologic Subtypes in the Matched Data

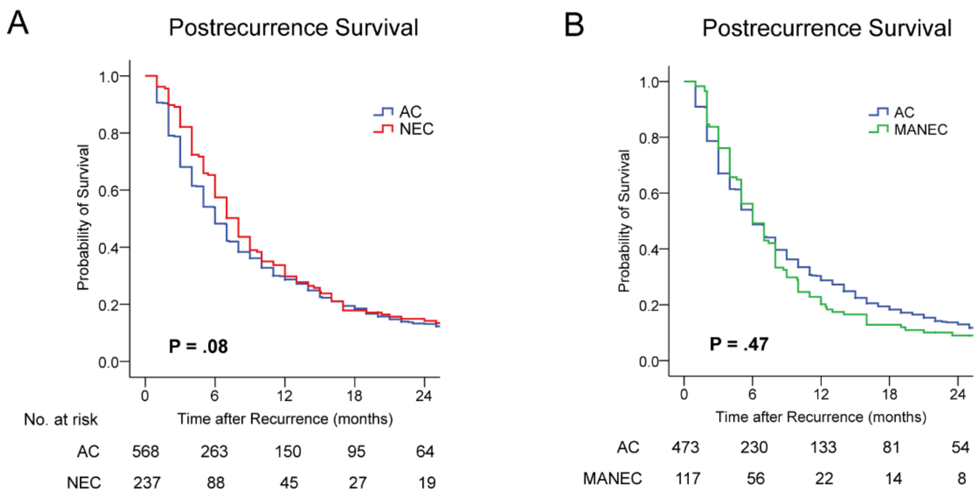

(A) gastric neuroendocrine carcinoma (NEC) versus adenocarcinoma (AC); (B) gastric mixed adenoneuroendocrine carcinoma (MANEC) versus AC.

© 2021 Lin J et al. *JAMA Network Open.*

eFigure 6. Kaplan-Meier Survival Curves for Patients With Gastric Neuroendocrine Carcinoma (NEC) and Adenocarcinoma (AC) in High-Volume Centers

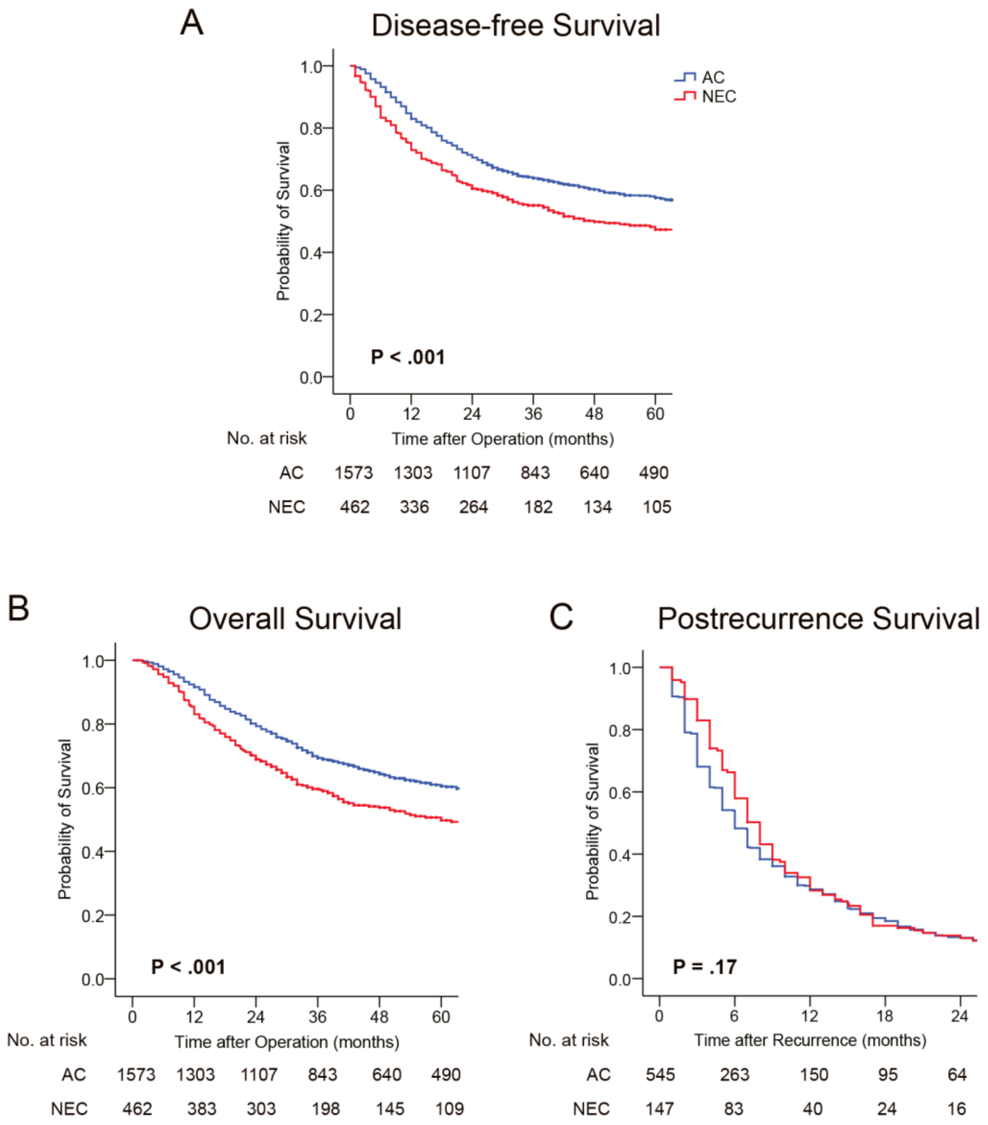

(A) Disease-free survival (DFS), (B) Overall survival (OS), and (C) postrecurrence survival (PRS).

© 2021 Lin J et al. *JAMA Network Open.*

eFigure 7. Recurrence Patterns According to Histologic Subtype in the Matched Data

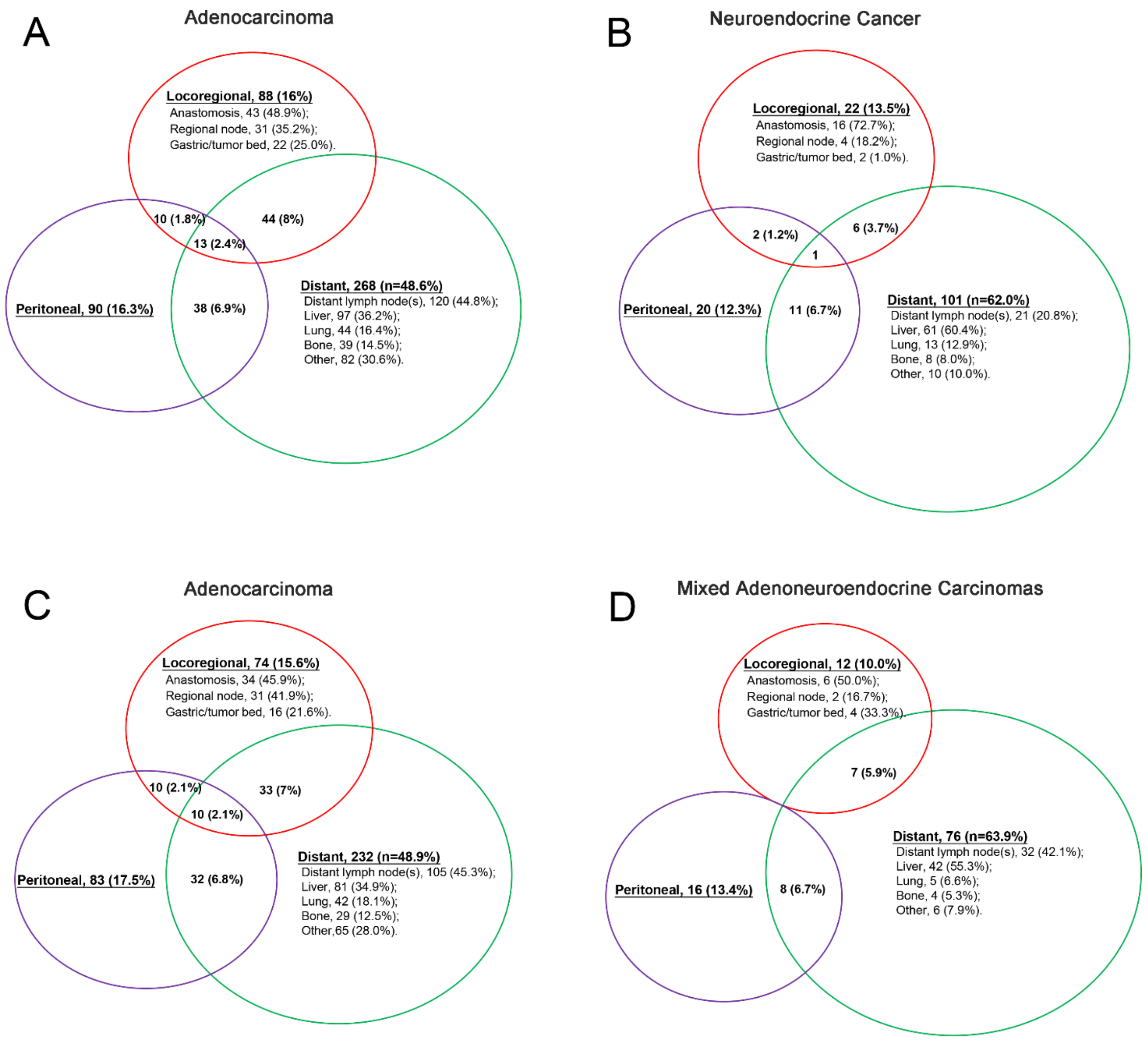

(A-B) matched adenocarcinoma and neuroendocrine carcinoma patients; (C-D) matched adenocarcinoma and mixed adenoneuroendocrine carcinoma patients. Sizes of circles are proportional to the number of patients.

© 2021 Lin J et al. *JAMA Network Open.*

eFigure 8. Kaplan-Meier Survival Curves for Patients With Gastric Mixed Adenoneuroendocrine Carcinoma (MANEC) and Adenocarcinoma (AC) in High-Volume Centers

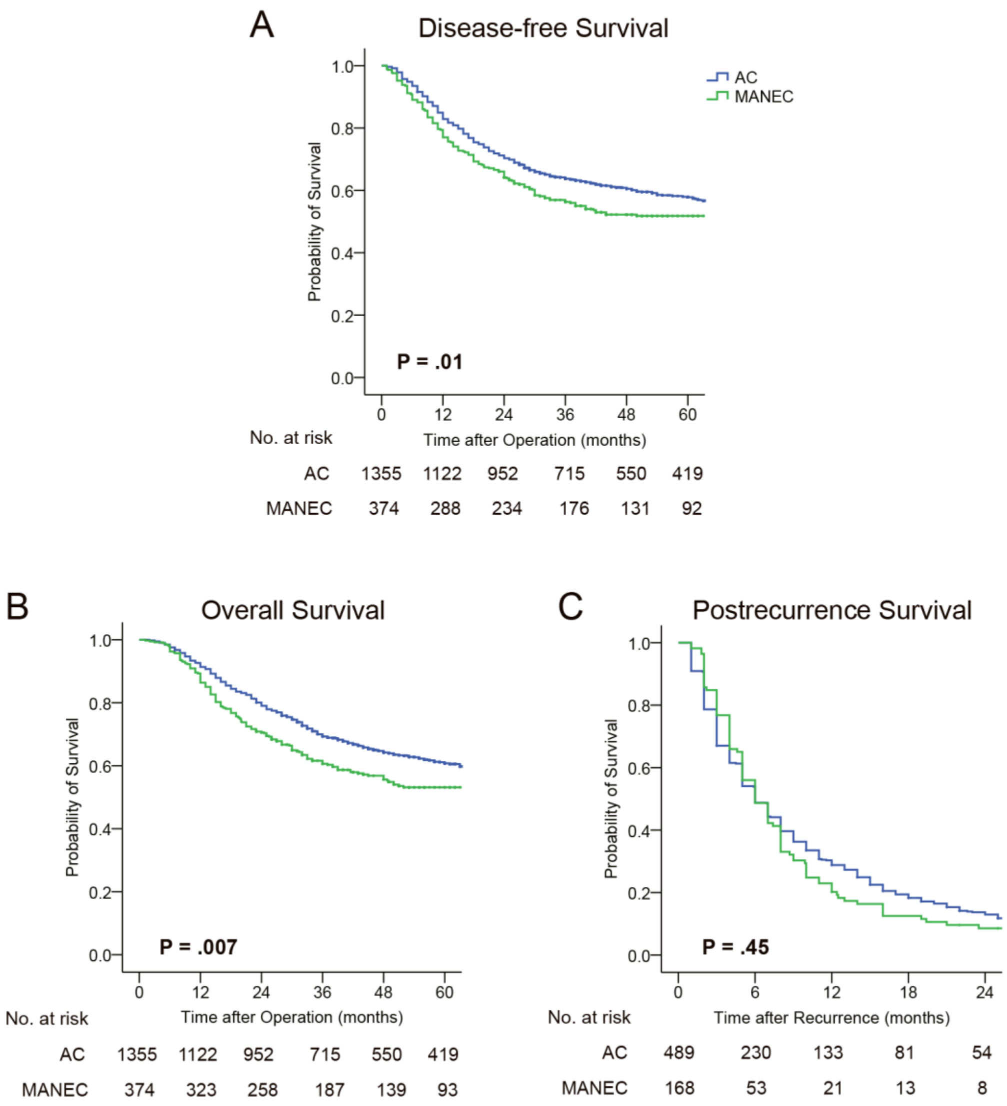

(A) Disease-free survival (DFS), (B) Overall survival (OS), and (C) postrecurrence survival (PRS).

© 2021 Lin J et al. *JAMA Network Open.*
